# Supplementary material for: Stacking Model‐Based Classifiers for Dealing With Multiple Sets of Noisy Labels
Source: Biom J. 2025 Mar 12;67(2):e70042. doi: 10.1002/bimj.70042 (PMC11898607; doi:10.1002/bimj.70042)
Supplement: Supplementary file 1 — Supporting Information [file BIMJ-67-e70042-s001.zip › density-based-ensemble-model-main/competitors_real_data/netlab-master/netlab-master/help/histp.htm]

Netlab Reference Manual histp


# histp

## Purpose

Histogram estimate of 1-dimensional probability distribution.

## Synopsis

```
h = histp(x, xmin, xmax, nbins)
```

## Description

`histp(x, xmin, xmax, nbins)` takes a column vector `x`
of data values and generates a normalized histogram plot of the
distribution. The histogram has `nbins` bins lying in the
range `xmin` to `xmax`.

`h = histp(...)` returns a vector of patch handles.

## See Also

`demgauss`

---

**Pages:**
Index


---

Copyright (c) Ian T Nabney (1996-9)
